# TFAP2C drives cisplatin resistance in bladder cancer by upregulating YAP and activating β-catenin signaling

**DOI:** 10.1016/j.jbc.2025.110387

**Published:** 2025-06-18

**Authors:** Qiufeng Pan, Changmin Zou, Zhigen Lin, Hao Tang, Zepu Long, Longwang Wang

**Affiliations:** 1 Department of Urology, The First Affiliated Hospital, Jiangxi Medical College, Nanchang University, Nanchang, China; 2 Department of Urology, Taihe County People's Hospital, JiAn, China; 3 The First Clinical Medical College, Nanchang University, Nanchang, China

**Keywords:** bladder cancer, cisplatin resistance, transcription regulation, YAP, β-catenin

## Abstract

Cisplatin-based chemotherapy is a conventional therapy for muscle-invasive bladder cancer (BC); however, its efficacy is often limited by the emergence of resistance to cisplatin. Yes-associated protein (YAP) and **β**-catenin are involved in this resistance, yet their upstream regulators are not well defined. This study investigates the role of TFAP2C in regulating YAP expression and its impact on cisplatin resistance in BC. The Cancer Genome Atlas (TCGA) gene expression data and GSE231835 dataset were analyzed to identify potential transcription factors regulating YAP. Assessed TFAP2C and YAP expression in clinical samples and cell lines. Functional assays were performed following TFAP2C knockdown. Dual-luciferase reporter assays and Chromatin immunoprecipitation (ChIP) confirmed TFAP2C binding to the YAP promoter. A mouse model evaluated the effects of TFAP2C silencing on tumor growth and cisplatin resistance. The results showed that TFAP2C was identified as an upstream activator of YAP, with elevated expression in cisplatin-resistant BC cell lines and positive correlation with YAP expression. Silencing TFAP2C reduced malignant behaviors, decreased YAP, phosphorylated YAP (p-YAP) and **β**-catenin levels, and increased apoptosis in both cisplatin-sensitive and cisplatin-resistant BC cells. Besides, TFAP2C directly binds to the YAP promoter, enhancing its transcription. In the xenograft model, TFAP2C silencing significantly inhibited tumor growth and reduced cisplatin resistance. TFAP2C promotes cisplatin resistance and malignant behavior in BC by upregulating YAP and activating the **β**-catenin signaling pathway. Targeting TFAP2C offers a novel therapeutic strategy to overcome cisplatin resistance in BC, representing a new discovery in combating chemoresistance.

Bladder cancer is the 10th most frequently diagnosed cancer worldwide, with approximately 573,000 new cases and 213,000 deaths reported in 2020, primarily affecting older individuals, particularly men older than 65 years of age (1, 2). Cisplatin-based chemotherapy remains the cornerstone for treating advanced bladder cancer; however, resistance to cisplatin is a significant clinical challenge, occurring in up to 50% of patients and resulting in therapeutic failure, high recurrence rates, and increased mortality (3). The mechanisms underlying cisplatin resistance are complex and include alterations in drug uptake and efflux, enhanced DNA repair processes, inhibition of apoptosis, and significant epigenetic changes (4, 5, 6). These insights underscore the necessity for further exploration of molecular pathogenesis to develop novel diagnostic biomarkers and therapeutic targets.

Yes-associated protein (YAP) has been identified as a key mediator of cisplatin resistance in bladder cancer by promoting cell survival and enhancing DNA damage repair mechanisms. Ciamporcero *et al.* (2016) demonstrated that YAP activation protects urothelial carcinoma cells from cisplatin-induced DNA damage, suggesting that targeting YAP could enhance chemotherapy efficacy (7). Further research by Ciamporcero *et al.* (2018) revealed a crosstalk between YAP and the Nrf2 pathway, where YAP influences the antioxidant response, contributing to chemoresistance (8). Additionally, Daga *et al.* (2019) demonstrated that the natural compound Ailanthone suppresses the growth and migration of cisplatin-resistant bladder cancer cells by downregulating the expression of YAP, Nrf2, and c-Myc, emphasizing the critical role of YAP in resistance mechanisms (9). Although these studies shed light on YAP's involvement in cisplatin resistance, the underlying mechanisms are not yet fully understood, indicating a need for more in-depth research in this area.

Recent research has revealed that epigenetic modifications, such as DNA methylation, histone modifications, and noncoding RNA regulation, play a pivotal role in driving chemoresistance (10, 11, 12). Advancements in understanding the epigenetic landscape of bladder cancer may provide a way for innovative strategies to overcome cisplatin resistance, ultimately improving survival rates and reducing the global burden of this disease.

## Results

### Expression and survival analysis of YAP in BC

**Figure 1:**
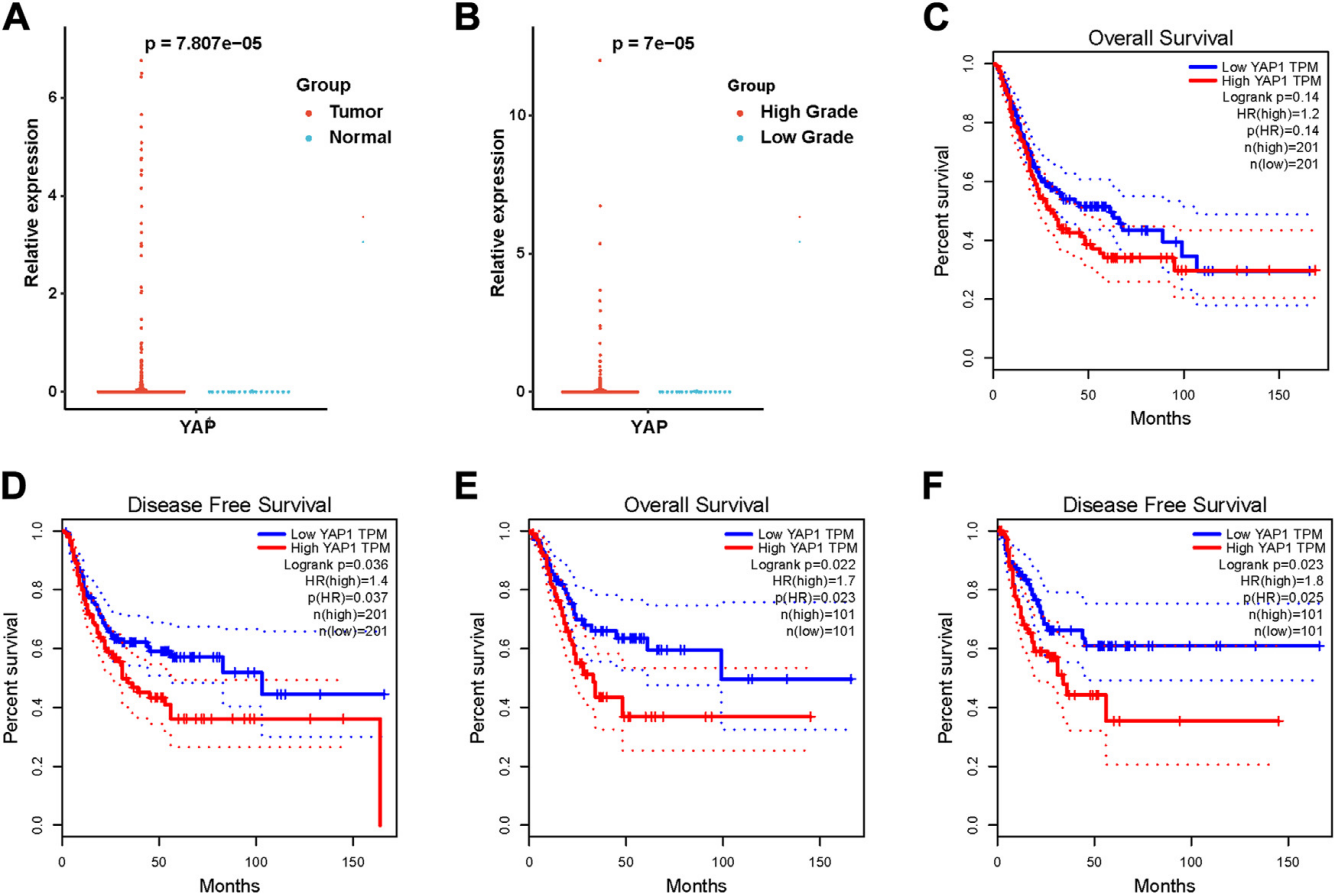
**Expression and survival analysis of YAP in BC**. *A*, expression of YAP in TCGA-BLCA in normal and BC tissues. *B*, expression of YAP in TCGA-BLCA in low and high grade BC tissues. OS (*C*) and DFS (*D*) of YAP in TCGA-BLCA, using median as cutoff. OS (*E*) and DFS (*F*) of YAP in TCGA-BLCA, using quartiles as cutoffs.

YAP expression and prognosis in TCGA-BLCA samples were analyzed by GEPIA (http://gepia.cancer-pku.cn/index.html) to examine its potential role in BC progression and prognosis. The results revealed that YAP expression was significantly higher in BC than in normal tissues (Fig. 1*A*). Furthermore, when stratified by tumor grade, YAP expression was higher in high-grade BC tissues relative to low-grade BC tissues, suggesting a possible correlation between YAP expression and tumor aggressiveness (Fig. 1*B*). On the other hand, when the median YAP expression was used as the cutoff, no significant difference in overall survival (OS) was observed between the high and low YAP expression groups (Fig. 1*C*). However, high YAP expression was significantly associated with a shorter disease-free survival (DFS) (Fig. 1*D*), indicating that elevated YAP expression may contribute to disease recurrence. Further analyses based on quartile stratification revealed a clearer pattern: patients with low YAP expression exhibited significantly longer OS and DFS compared to those with high YAP expression (Fig. 1, *E* and *F*). This suggests that high YAP expression may serve as a prognostic marker associated with worse outcomes in BC patients, highlighting YAP as a potential indicator of poor prognosis.

### Differential gene analysis in the BC resistance dataset

**Figure 2:**
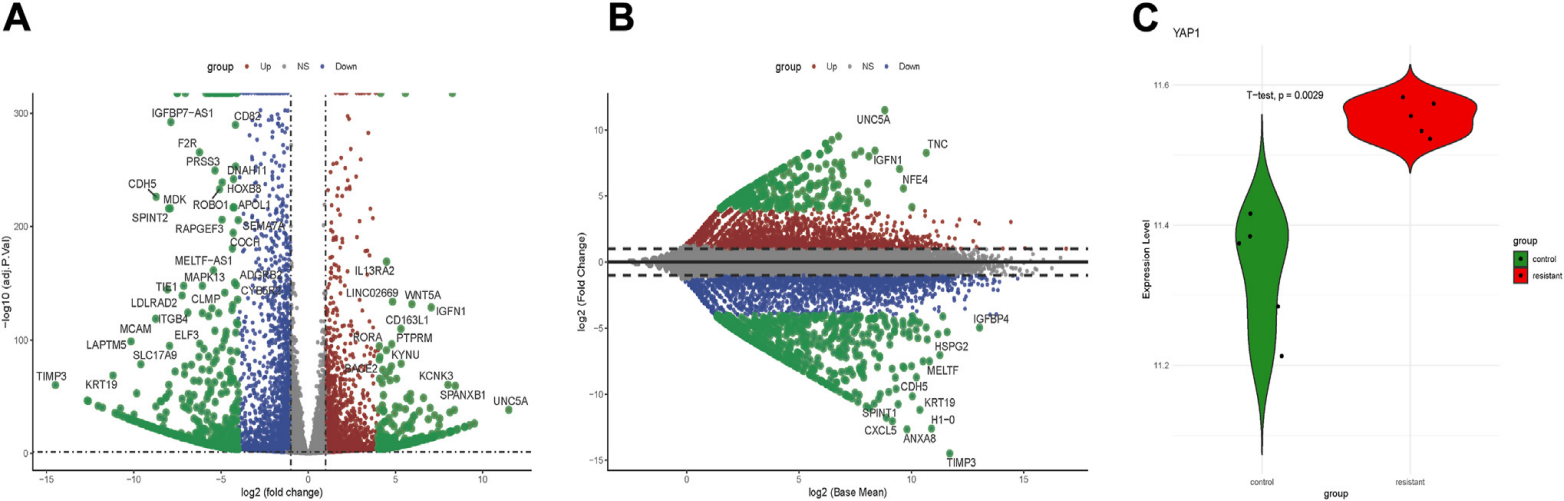
**Differential gene analysis of the BC resistance dataset GSE231835.** Volcano plot (*A*) and MA plot (*B*) showing differentially expressed genes in the GSE231835 dataset. *C*, Expression of YAP in the GSE231835 dataset. Differential genes were screened using an absolute value of *p* < 0.05 and | log2FC | >1 as the threshold.

To investigate potential mechanisms of chemoresistance in bladder cancer, differential gene expression analysis was performed on the GSE231835 dataset, which includes sequencing data from the bladder cancer cell line T24 and its cisplatin-resistant derivative, T24R2. Genes with a *p*-value < 0.05 and |log2FC| > 1 were selected as differentially expressed genes (DEGs), resulting in a total of 5667 DEGs between the resistant and control cell lines. The volcano plot (Fig. 2*A*) and MA plot (Fig. 2*B*) visually illustrate the distribution of these DEGs, with significant upregulation or downregulation in T24R2 cells. Notably, the expression level of YAP was significantly elevated in the T24R2 resistant cell line compared to the parental T24 line (Fig. 2*C*), suggesting a potential role of YAP in cisplatin resistance.

This observation aligns with the survival analysis results shown in Figure 1, where higher YAP expression was associated with poorer prognosis in bladder cancer patients. The increased YAP expression in the resistant cell line further supports its potential involvement in resistance mechanisms, positioning YAP as a candidate marker for chemoresistance and as a potential target for therapeutic intervention in bladder cancer.

### Identification of YAP-related upstream transcription factors (TFs)

**Figure 3:**
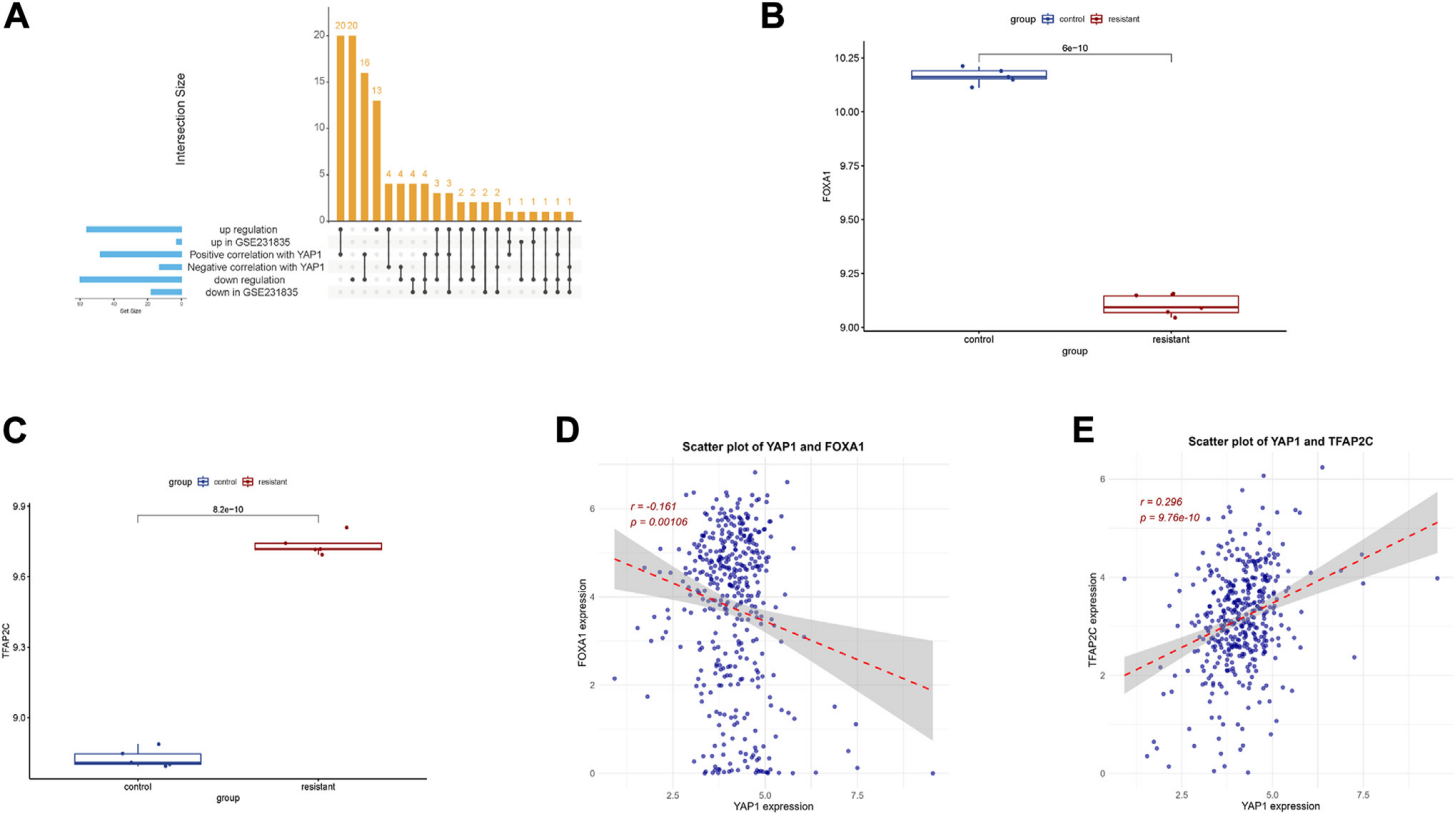
**Identification of YAP-related upstream TFs.***A*, upset plot showing identification of potential TFs for YAP in the integrated KnockTF database and GSE231835 dataset. *B*, FOXA1 expression in the GSE231835 dataset. *C*, TFAP2C expression in the GSE231835 dataset. *D*, Pearson correlation analysis of FOXA1 with YAP in the TCGA-BLCA dataset. *E*, Pearson correlation analysis of TFAP2C with YAP in the TCGA-BLCA dataset.

To identify potential upstream TFs regulating YAP, an integrative analysis was conducted using the KnockTF v2 database and the GSE231835 dataset. A total of 125 regulatory interactions were identified, involving 105 TFs as potential regulators of YAP (Fig. 3*A*). Among these, 20 TFs had both upregulation and downregulation effects on YAP upon knockout, 42 TF knockouts resulted in YAP downregulation, and 43 knockouts led to YAP upregulation, indicating complex regulatory relationships. Using the TCGA-BLCA dataset from UCSC Xena, Pearson correlation analysis was performed to assess the expression relationship between YAP and its upstream TFs in bladder urothelial carcinoma (BLCA) samples. Of the 105 TFs identified, 61 showed significant correlations with YAP, with 48 positively correlated and 13 negatively correlated (Table S2). Additionally, differential expression analysis of these TFs in the GSE231835 dataset revealed that 3 TFs were significantly upregulated, while 18 were significantly downregulated in the cisplatin-resistant samples compared to controls (Table S3).

The Upset plot in Figure 3*A* highlights the intersection of TFs across datasets, with FOXA1 and TFAP2C selected for further analysis due to their notable associations with YAP. FOXA1 was observed to be downregulated in the GSE231835 dataset's cisplatin-resistant samples (Fig. 3*B*) and showed a significant negative correlation with YAP expression in the TCGA-BLCA dataset (Fig. 3*D*). According to the KnockTF database, silencing FOXA1 can lead to both upregulation and downregulation of YAP, suggesting that FOXA1 may have context-dependent regulatory effects on YAP expression.

TFAP2C, on the other hand, exhibited a significant positive correlation with YAP expression in the TCGA-BLCA dataset (Fig. 3*E*). TFAP2C was significantly upregulated in the cisplatin-resistant GSE231835 samples (Fig. 3*C*), aligning with the KnockTF database's findings where silencing FOXA1 led to an increase in YAP expression. These results suggest that TFAP2C may function as an activator of YAP in the context of cisplatin resistance, providing insights into the regulatory network influencing YAP and potentially contributing to chemoresistance mechanisms in bladder cancer.

### Expression and prognostic analysis of FOXA1 and TFAP2C in TCGA-BLCA

**Figure 4:**
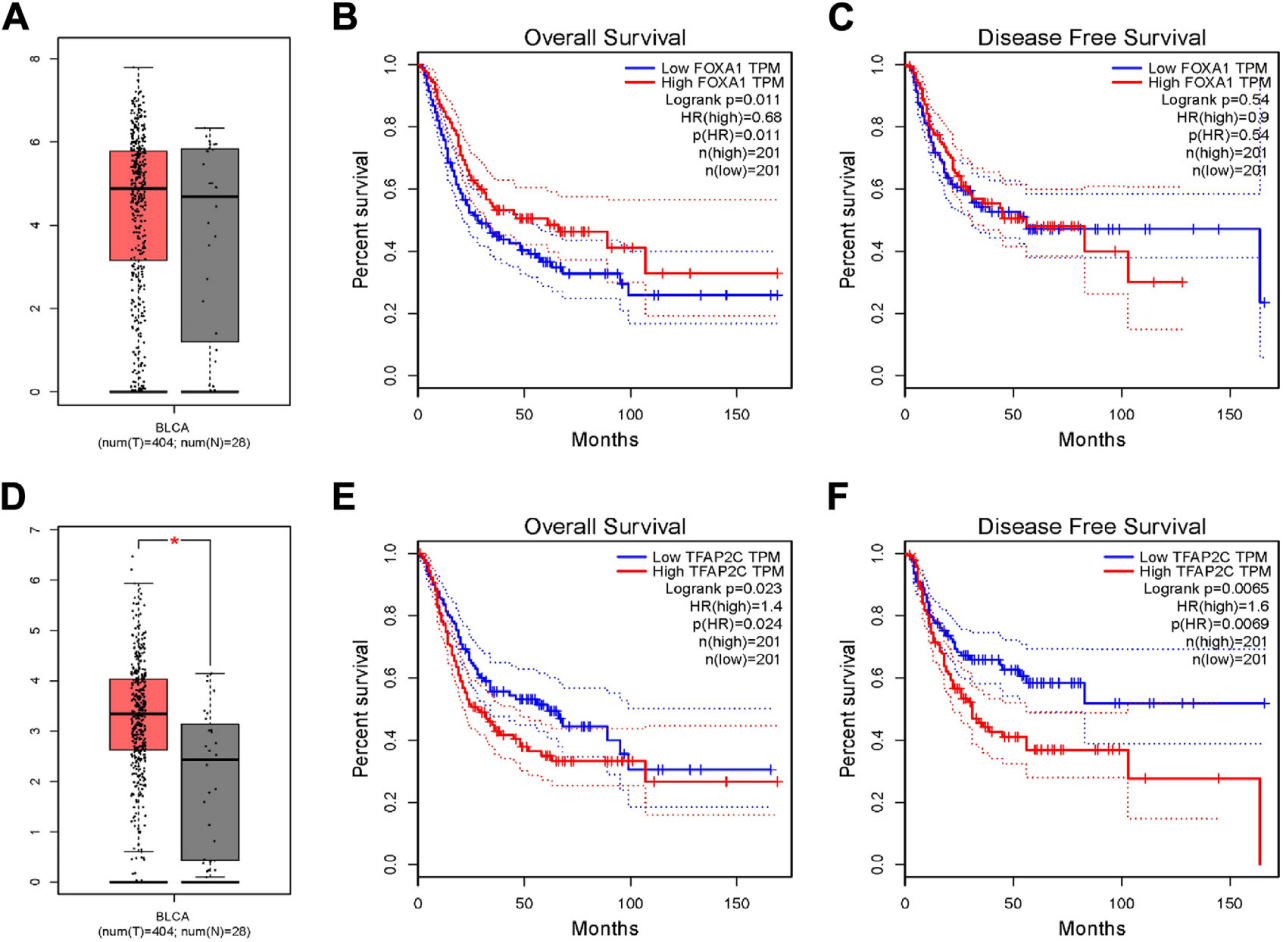
**Expression and prognostic analysis of FOXA1 and TFAP2C in TCGA-BLCA.***A*, FOXA1 expression in TCGA-BLCA samples (*Red* color represented the tumor samples, *Black* color represented the normal samples). OS (B) and DFS (C) of FOXA1 in TCGA-BLCA. *D*, TFAP2C expression in TCGA-BLCA samples (*Red* color represented the Tumor samples, *Black* color represented the Normal samples). OS (*E*) and DFS (*F*) of TFAP2C in TCGA-BLCA.

To investigate the differential expression and prognostic relevance of FOXA1 and TFAP2C in BC, data from TCGA-BLCA samples were analyzed using GEPIA (http://gepia.cancer-pku.cn/index.html), applying a threshold of *p* < 0.05 and | log2FC | >1. The analysis revealed that FOXA1 expression showed no significant difference between tumor and normal control samples (Fig. 4*A*), while TFAP2C expression was significantly higher in BLCA tumor samples (Fig. 4*D*). This upregulation of TFAP2C in cancer samples suggests its potential involvement in tumor progression.

Survival analysis further examined the association between FOXA1 and TFAP2C expression levels and patient outcomes. High FOXA1 expression was significantly correlated with longer OS in patients with BLCA, although it was not associated with DFS (Fig. 4, *B* and *C*). In contrast, high TFAP2C expression was linked to significantly poorer outcomes in both OS and DFS (Fig. 4, *E* and *F*). These findings suggest that elevated TFAP2C levels could exacerbate tumor malignancy, aligning with their distinct regulatory effects on YAP expression and involvement in drug resistance mechanisms.

In summary, FOXA1 and TFAP2C appear to have opposing roles in BLCA, with FOXA1 associated with longer survival and TFAP2C serving as a marker for poor prognosis. These results further support the role of TFAP2C as an upstream activator of YAP in mediating drug resistance in cancer, highlighting its potential as a therapeutic target for BLCA.

### TFAP2C Promotes Malignant Behavior and Stemness in BC

**Figure 5:**
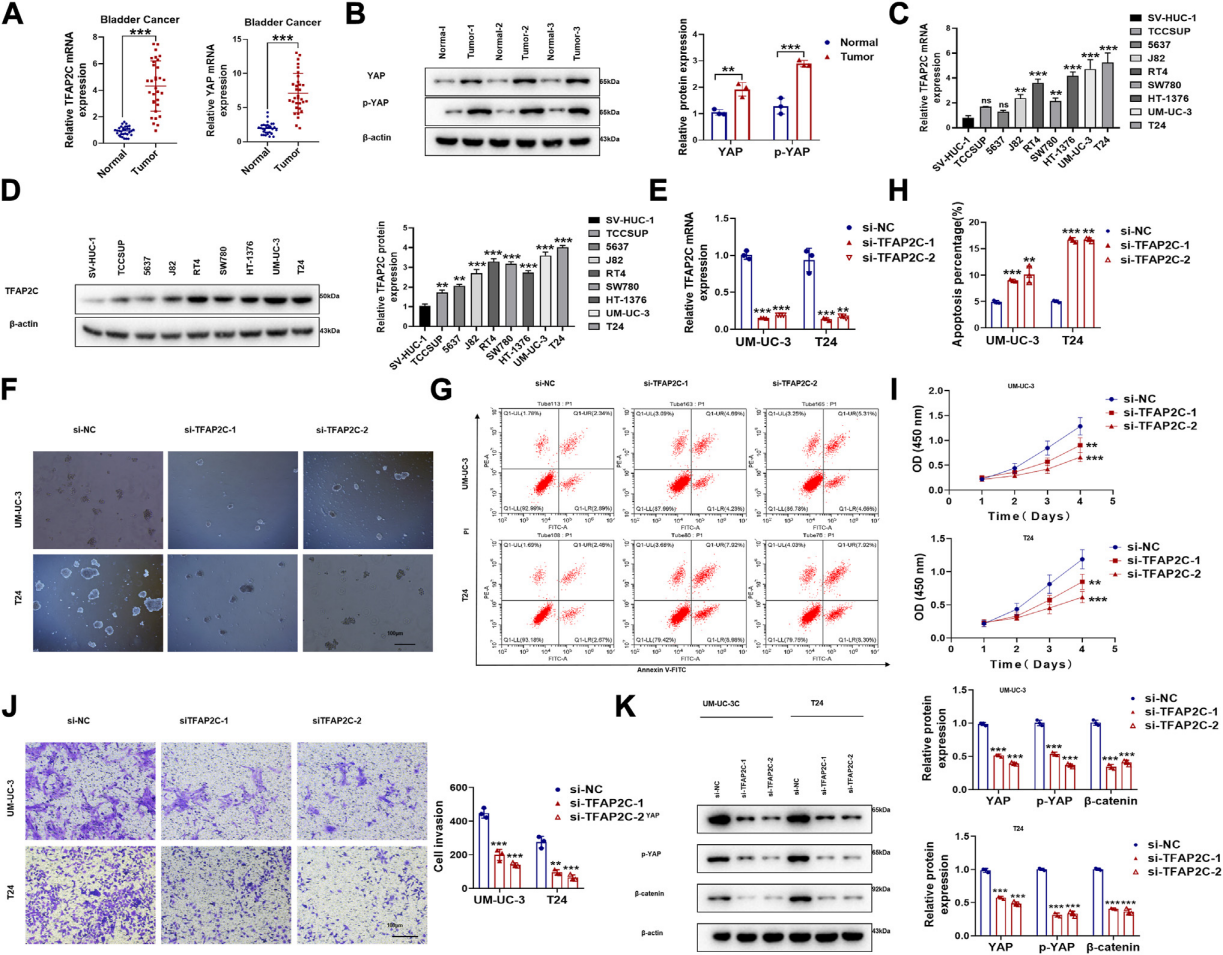
**TFAP2C Promotes Malignant Behavior and Stemness in BC.***A*, mRNA levels of TFAP2C and YAP in 32 BC patients' cancer tissue samples and their adjacent normal tissue samples.(n = 32). *B*, Protein levels of YAP and p-YAP expression in 32 BC patients' cancer tissue samples and their adjacent normal tissue samples. *C*, mRNA and (*D*) protein expression of TFAP2C in immortalized normal human bladder epithelial cell line SV-HUC-1 and eight bladder cancer cell lines (T24, 5637, J82, RT4, UM-UC-3, SW780, HT-1376, TCCSUP). *E*, Confirmation of TFAP2C knockdown in UM-UC-3 and T24 cells by qRT-PCR. *F*, sphere formation assay (scale bar = 100 μm) and (*G*) apoptosis assay in UM-UC-3 and T24 cells after TFAP2C knockdown. *H*, quantitative analysis of apoptosis rates. *I*, CCK-8 assay and (*J*) transwell invasion assay in TFAP2C-silenced UM-UC-3 and T24 cells. (scale bar = 100 μm), (*K*) Protein levels of YAP, p-YAP, and β-catenin in UM-UC-3 and T24 cells after TFAP2C knockdown. N = 3 biologically independentsamples. “ns” indicates non-significant, ∗∗*p* < 0.01; ∗∗∗*p* < 0.001.

To understand the expression and clinical significance of TFAP2C and YAP in BC, we first analyzed the mRNA and protein levels in 32 BC tissues and adjacent normal tissues. Both TFAP2C and YAP mRNA levels were significantly elevated in tumor samples (Fig. 5*A*), and Western blot confirmed higher YAP and p-YAP protein levels in BC tissues (Fig. 5*B*). Next, we examined TFAP2C expression levels across a range of BC cell lines and in the immortalized normal bladder epithelial cell line SV-HUC-1, which showed the lowest TFAP2C expression. In contrast, TFAP2C expression was progressively higher across BC cell lines, with notably elevated levels in T24 and UM-UC-3 cells, suggesting an association between TFAP2C expression and cancerous characteristics (Fig. 5, *C* and *D*).

To further investigate TFAP2C's functional role, we performed TFAP2C knockdown in UM-UC-3 and T24 cells (Fig. 5*E*). Silencing TFAP2C resulted in significant reductions in sphere-forming ability, increased apoptosis, as well as a notable decrease in cell proliferation and invasion capacity (Fig. 5, *F*–*J*), supporting the hypothesis that TFAP2C promotes malignant biological behavior in BC cells. Moreover, Western blot analysis demonstrated that TFAP2C knockdown significantly reduced YAP and p-YAP expression levels, along with β-catenin expression (Fig. 5*K*), indicating that TFAP2C may regulate YAP and β-catenin signaling pathways. These findings suggest that TFAP2C contributes to the maintenance of stemness in BC cells, by modulating YAP and β-catenin expression, underscoring TFAP2C's role as a potential therapeutic target in BC.

### TFAP2C promotes stemness and cisplatin resistance in BC through YAP regulation

**Figure 6:**
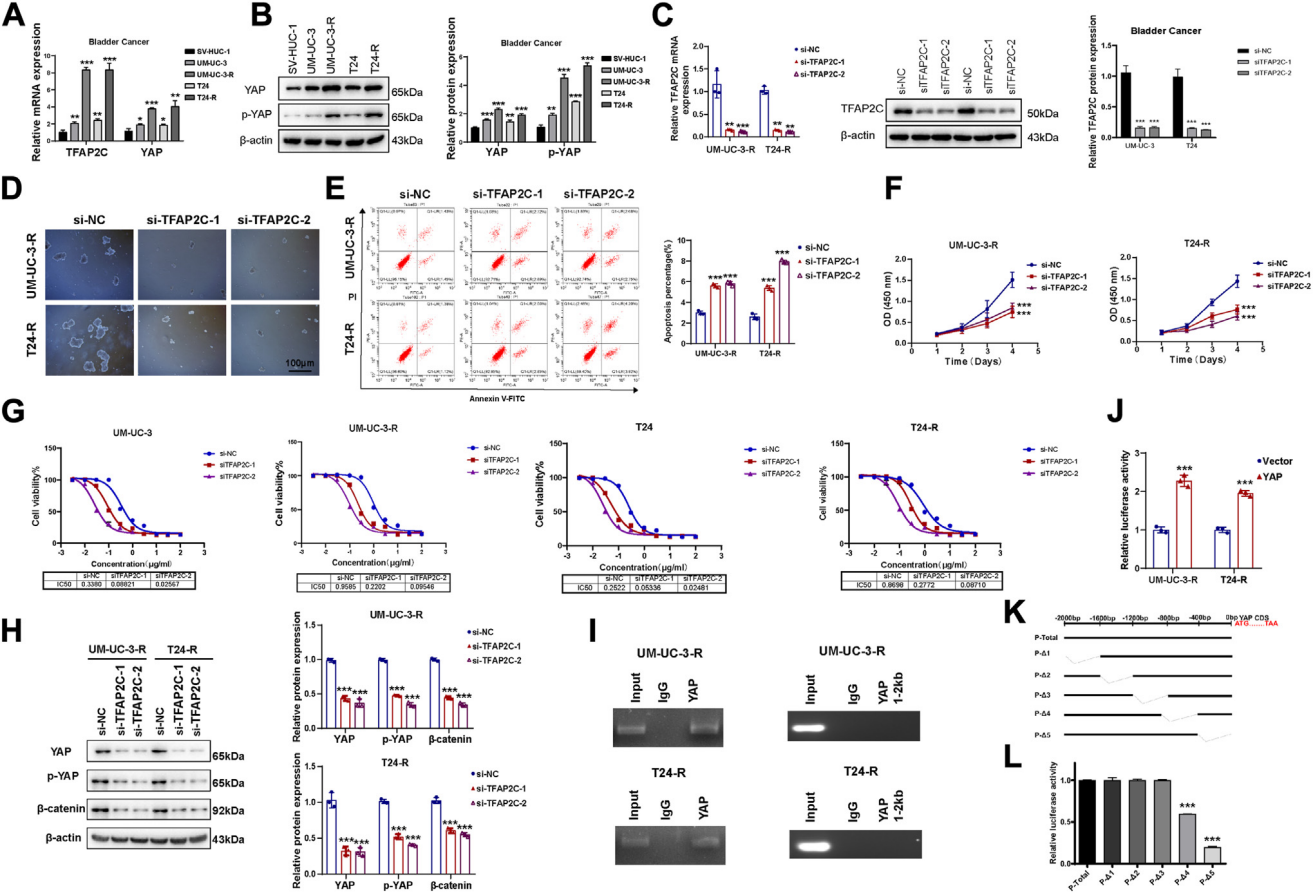
**TFAP2C Promotes Stemness in BC Cells by Regulating YAP and β-Catenin Pathways.***A*, relative mRNA levels of TFAP2C and YAP in UM-UC-3-R and T24-R cells. *B*, Protein levels of YAP and p-YAP in UM-UC-3-R and T24-R cells. *C*, efficiency of TFAP2C knockdown in UM-UC-3-R and T24-R cells confirmed by qRT-PCR and Western blots. *D*, sphere formation assay (scale bar = 100 μm), (*E*) apoptosis assay, (*F*) CCK-8 assay in TFAP2C-silenced UM-UC-3-R and T24-R cells. *G*, IC_50_ of cisplatin in TFAP2C-silenced UM-UC-3-R and T24-R cells compared to the parental cells. *H*, protein levels of YAP, p-YAP, and β-catenin in TFAP2C-silenced UM-UC-3-R and T24-R cells. *I*, ChIP-PCR analysis of TFAP2C binding to the YAP promoter region and a distal region (∼1–2 kb upstream of the transcription start site) in UM-UC-3-R and T24-R cells. Input and IgG were used as controls. *J*, Luciferase activity of the YAP promoter in UM-UC-3-R and T24-R cells co-transfected with YAP promoter-luciferase reporter and either empty vector (*blue*) or TFAP2C expression vector (*red*). *K*, schematic of YAP promoter deletion constructs (P-Δ1–P-Δ5) based on the −2000 to 0 bp region upstream of the YAP coding sequence. *L*, Luciferase activity of each construct co-transfected with TFAP2C in 293T cells. n = 3 biologically independentsamples. ∗*p* < 0.05; ∗∗*p* < 0.01; ∗∗∗*p* < 0.001.

To investigate the role of TFAP2C in cisplatin-resistant BC, we established cisplatin-resistant BC cell lines, UM-UC-3-R and T24-R. Quantitative analysis showed that mRNA expression of TFAP2C was significantly upregulated in these resistant cell lines and that mRNA and protein levels of YAP were also elevated (Fig. 6, *A* and *B*). We silenced TFAP2C in UM-UC-3-R and T24-R cells to further examine its function (Fig. 6*C*). Functional assays showed that TFAP2C knockdown significantly reduced sphere formation, indicating a decrease in stemness (Fig. 6*D*), and increased apoptosis in resistant cells (Fig. 6*E*). CCK-8 assays demonstrated a marked reduction in cisplatin resistance in TFAP2C-silenced cells, with decreased cisplatin IC50 values in both UM-UC-3/2nd and T24/2nd cells (Fig. 6, *F* and *G*).

Western blot analysis demonstrated that TFAP2C knockdown led to a reduction in the expression of YAP, p-YAP, and β-catenin proteins, indicating a connection between TFAP2C and these pathways in mediating drug resistance in BC (Fig. 6*H*). ChIP assays demonstrated significant enrichment of TFAP2C at the YAP promoter in UM-UC-3-R and T24-R cells compared to IgG controls, while no enrichment was observed at a distal region ∼1.8 to 2 kb upstream of the transcription start site, confirming binding specificity (Fig. 6*I*). Furthermore, cells were co-transfected with a YAP promoter-luciferase construct and either an empty vector or a TFAP2C expression plasmid, and luciferase activity measured 48 h later was significantly higher in the TFAP2C group compared to the control (Fig. 6*J*). These results highlight that in cisplatin-resistant BC cells, TFAP2C acts as a transcription factor that binds to the YAP promoter, enhancing YAP expression and contributing to resistance. Targeting TFAP2C may represent a promising therapeutic strategy to overcome cisplatin resistance and reduce stemness in BC. To identify the key TFAP2C-responsive region within the YAP promoter, we generated a series of deletion luciferase reporters spanning the −2000 to 0 bp region (Fig. 6*K*). Co-transfection with TFAP2C revealed that deletion of the −800 to 0 bp region, especially −400 to 0 bp (P-Δ5), led to a marked decrease in promoter activity (Fig. 6*L*). These results, consistent with ChIP and luciferase assays, indicate that TFAP2C activates YAP transcription by binding to its proximal promoter.

**Figure 7:**
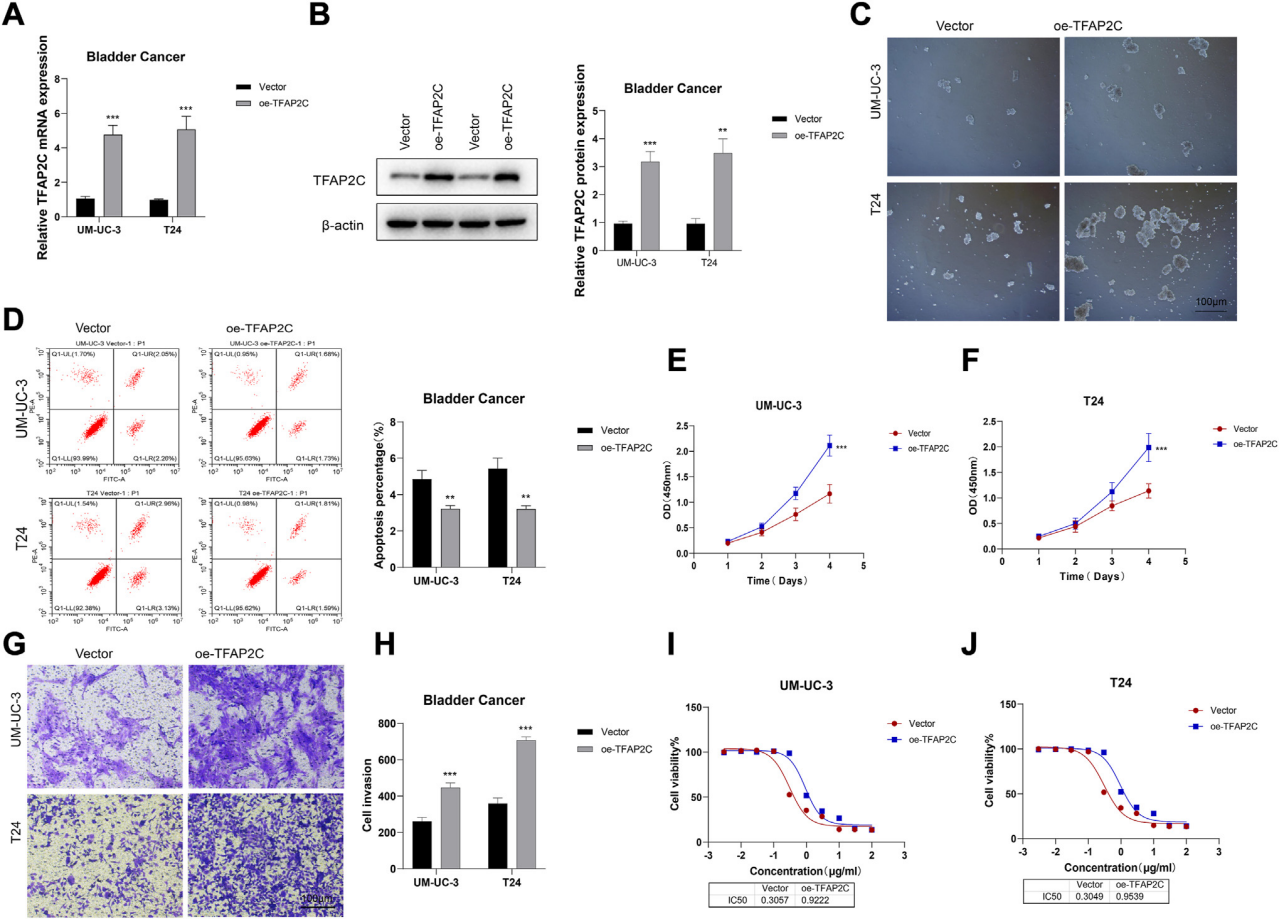
**TFAP2C drives cisplatin resistance and cancer stemness by binds to the YAP promoter.***A and B*, efficiency of TFAP2C overexpression in UM-UC-3 and T24 cells confirmed by qRT-PCR and Western blots. *C*, sphere formation assay (scale bar = 100 μm), (*D*) apoptosis assay, (*E and F*) CCK-8 assay in TFAP2C-overexpressed UM-UC-3 and T24 cells. *G and H*, transwell invasion assay in TFAP2C-overexpressed UM-UC-3 and T24 cells. (scale bar = 100 μm). *I and J*, IC50 of cisplatin in TFAP2C-overexpressed UM-UC-3-R and T24-R cells. ∗∗*p* < 0.01; ∗∗∗*p* < 0.001.

To further confirm that TFAP2C drives cisplatin resistance and cancer stemness by binding to the YAP promoter, we added experiments with exogenous overexpressing TFAP2C to explore its effect on cisplatin sensitivity and cellular phenotype (Fig. 7). The TFAP2C overexpression cell line was constructed and confirmed by qRT-PCR (Fig. 7, *A* and *B*). Next, the sphere formation Assay showed that overexpressing TFAP2C significantly increased sphere formation, indicating an increase in stemness of cancer cells (Fig. 7*C*). Besides, overexpression of TFAP2C decreases the apoptosis percentage in resistant cells (Fig. 7*D*) and increases cell proliferation (Fig. 7, *E* and *F*). Overexpressing TFAP2C were also increased the ability of the transfer and invasion in resistant cells (Fig. 7, G-H). Finally, the result of IC50 showed that overexpressing TFAP2C can induce cisplatin resistance (Fig. 7, *I* and *J*). These results further strengthen the conclusion that TFAP2C promotes stemness and cisplatin resistance in BC through YAP regulation.

### Effect of TFAP2C on tumor growth and cisplatin resistance in BC xenograft model

**Figure 8:**
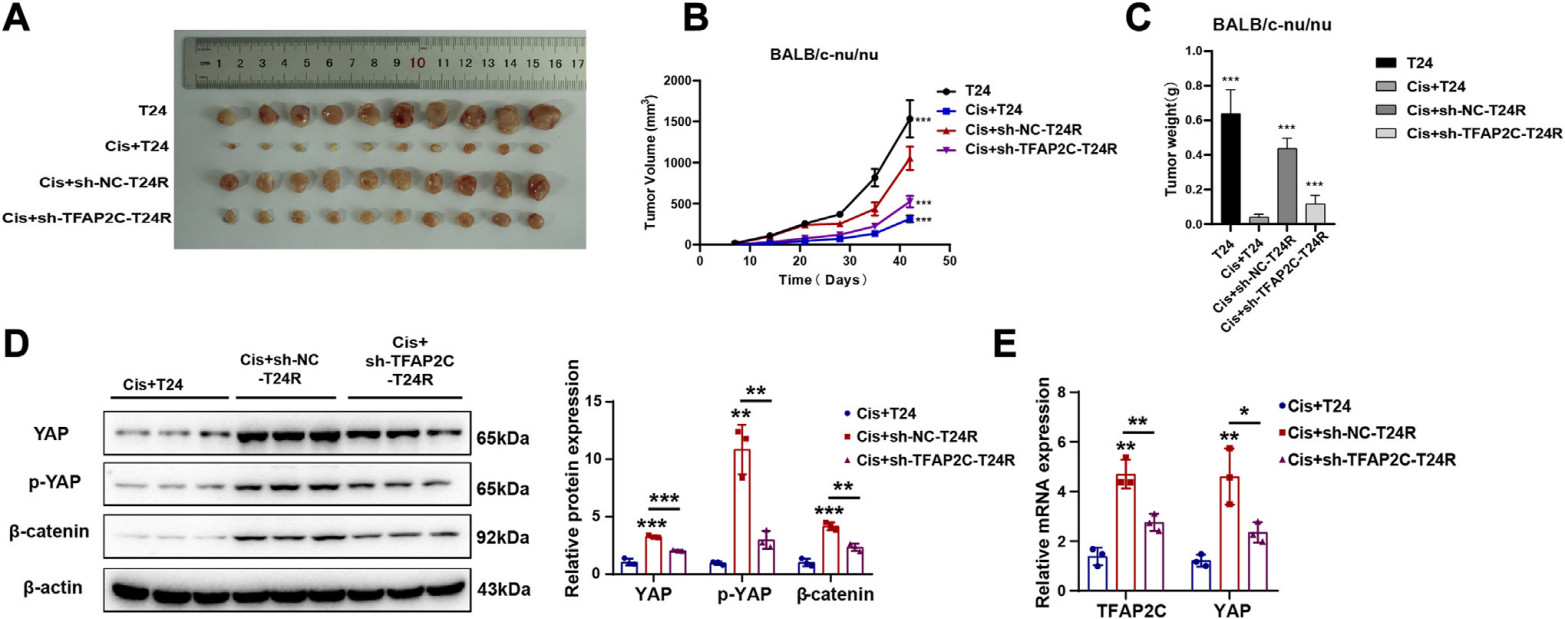
**Effect of TFAP2C on tumor growth and cisplatin resistance in BC xenograft model**. *A*, representative images of tumors excised from each group of mice. *B*, tumor growth curves showing mean tumor volume (mm^3^) over time for each group (n = 10 per group). *C*, final tumor weights measured at the endpoint. *D*, protein expression levels of YAP, p-YAP, and β-catenin in tumor tissues from each group. *E*, mRNA expression levels of YAP and TFAP2C in tumor tissues from each group. ∗*p* < 0.05; ∗∗*p* < 0.01; ∗∗∗*p* < 0.001.

To investigate the effects of TFAP2C on BC xenograft growth and cisplatin resistance, we used 6-week-old female nude mice to establish a xenograft model (BALB/c-nu/nu). The mice were divided equally into 3 groups: cisplatin-sensitive, cisplatin-resistant, and TFAP2C-silenced cisplatin-resistant groups. Subcutaneous tumor models were established using tumour cells (T24 or T24-R) and treated with cisplatin to assess tumor growth and drug resistance. According to the experimental results, the tumors in the cisplatin-resistant group were significantly larger compared to those in the cisplatin-sensitive group, while tumor growth was markedly slowed in the TFAP2C-silenced cisplatin-resistant group (Fig. 8, *A*–*C*). These findings suggest that silencing TFAP2C effectively inhibits tumor growth in cisplatin-resistant bladder cancer.

Western blot analysis revealed that the protein expression levels of YAP, p-YAP, and β-catenin were significantly higher in the cisplatin-resistant group, but were notably reduced in the TFAP2C-silenced cisplatin-resistant group (Fig. 8*D*). This suggests that TFAP2C silencing may regulate cisplatin resistance through modulation of YAP and β-catenin signaling. Additionally, qRT-PCR analysis showed that mRNA levels of both YAP and TFAP2C were significantly elevated in the cisplatin-resistant group, whereas silencing TFAP2C resulted in a marked decrease in their expression (Fig. 8*E*), further supporting the role of TFAP2C in regulating these pathways.

The results highlight the tumorigenic potential of TFAP2C *in vivo*, and targeting TFAP2C and its associated signaling pathways could offer potential therapeutic value in overcoming drug resistance.

## Discussion

Bladder cancer is a common malignancy worldwide, with cisplatin-based chemotherapy being a primary treatment for muscle-invasive urothelial carcinoma. However, the effectiveness of cisplatin is often limited due to the development of resistance (7). Recent studies have identified Yes-associated protein (YAP) and β-catenin as key players in mediating this resistance. YAP, a crucial component of the Hippo signaling pathway, has been shown to protect bladder cancer cells from cisplatin-induced DNA damage by enhancing DNA repair mechanisms and promoting cell survival (13). Hydroxynonenal has been reported to post-translationally downregulate YAP *via* a redox-dependent mechanism, contributing to its specific anticancer effects, including the inhibition of angiogenesis, accumulation of cells in the G2/M phase, and induction of apoptosis (14). Overexpression of YAP correlates with increased chemoresistance, while its inhibition sensitizes cells to cisplatin (7). Additionally, YAP and nuclear factor erythroid 2–related factor 2 (Nrf2) interaction influencing antioxidant responses that contribute to resistance (8). Daga *et al.* (2019) highlights that Ailanthone restrains the growth of cisplatin-resistant bladder cancer cells by downregulating YAP, Nrf2, and c-Myc expressions (9). Similarly, Wnt/β-catenin signaling pathway acts a vital role in cisplatin resistance. Chen *et al.* (2021) demonstrated that SC66 led to apoptosis and inhibits proliferation in bladder cancer cells by targeting the AKT/β-catenin pathway, indicating β-catenin's contribution to cell survival and chemoresistance (15). Li *et al.* (2021) reported that magnesium supplementation modulating the Wnt/β-catenin signaling pathway and then enhances cisplatin's inhibitory effect on bladder cancer cell survival, leading to decreased nuclear β-catenin levels and increased apoptosis (16). Furthermore, Shi *et al.* (2024) showed that LASS2 enhances chemosensitivity to cisplatin by inhibiting PP2A-mediated β-catenin dephosphorylation in stem-like bladder cancer cells, thereby reducing the transcription of genes associated with drug resistance (17). Ecn has been validated to inhibit tumor growth by activating p38 and suppressing Wnt/β-catenin pathways, and when combined with cisplatin or gemcitabine, it exhibits synergistic inhibitory effects on bladder cancer cells (18). Despite these advances, the precise mechanisms by which YAP and β-catenin contribute to cisplatin resistance in bladder cancer are not yet fully elucidated, highlighting the need for further in-depth research to develop targeted therapeutic strategies. The transcription factor family activator protein 2 (TFAP2) is vital for regulating oncogenic development (19).Several studies have reported feasible potential ways of targeting TFAP2C proteins, such as designing the DNA Aptamers/Oligonucleotides and direct binding to TFAP2C's DNA-binding domain, blocking gene activation (20), Utilizing the PROTACs (proteolysis-targeting chimeras) to remove disease-causing proteins selectively (21) and so on.

This study investigated the mechanisms of cisplatin resistance in bladder cancer (BC) by integrating bioinformatics analyses with experimental validation. Initially, differential expression analysis using the TCGA-BLCA dataset revealed that Yes-associated protein (YAP) was significantly upregulated in BC tissues, especially in high-grade tumors, and higher YAP expression correlated with poorer disease-free survival. Further analysis of the GSE231835 dataset, which compares cisplatin-resistant T24R2 cells to parental T24 cells, showed that YAP expression was notably increased in resistant cells, suggesting its role in chemoresistance.

To identify upstream regulators of YAP, the KnockTF database was utilized alongside Pearson correlation analysis in the TCGA-BLCA dataset. This integrative approach highlighted TFAP2C as a potential transcription factor that positively regulates YAP. Experimental validation confirmed that TFAP2C expression was significantly elevated in cisplatin-resistant BC cell lines (UM-UC-3-R and T24-R) and positively correlated with YAP expression. Silencing TFAP2C in these cells led to decreased YAP and β-catenin levels.

Functional assays demonstrated that TFAP2C knockdown reduced malignant behaviors, such as cell proliferation, invasion, and sphere formation (indicative of stemness), while increasing apoptosis. Chromatin immunoprecipitation, dual-luciferase reporter assays and exogenous overexpression of TFAP2C experiments confirmed that TFAP2C directly binds to the YAP promoter region, enhancing its transcription. *In vivo*, silencing TFAP2C in a cisplatin-resistant BC xenograft model resulted in significantly reduced tumor growth and decreased expression of YAP and β-catenin. The study combined bioinformatics and experimental methods to uncover that TFAP2C promotes malignant behavior and cisplatin resistance in BC by upregulating YAP and β-catenin signaling pathways. These findings suggest that targeting TFAP2C could be a promising strategy to overcome cisplatin resistance in bladder cancer patients.

Transcription factor AP-2 gamma (TFAP2C) plays a pivotal role in bladder cancer progression. It is overexpressed in high-grade bladder cancer tissues and is associated with poor clinical outcomes by promoting tumor growth, proliferation, and invasion (22). TFAP2C regulates genes involved in cell cycle progression and epithelial–mesenchymal transition, contributing to tumor aggressiveness (23).

In the context of cisplatin resistance, TFAP2C modulates chemotherapy sensitivity in bladder cancer cells. Xing *et al.* (2022) reported that knockdown of TFAP2C enhances the anti-tumor effects of cisplatin by inducing cell cycle arrest and apoptosis, as well as inhibiting migration and invasion. This effect is mediated through the regulation of EGFR and NF-κB signaling pathways, which are crucial for cell survival and chemoresistance (24).

Moreover, TFAP2C interacts with other molecular pathways influencing tumor progression and drug resistance. For instance, Kołat *et al.* (2021) showed that TFAP2C collaborates with the tumor suppressor WWOX to modulate signaling pathways related to bladder cancer aggressiveness (25). Wang *et al.* (2018) demonstrated that TFAP2C enhances stemness and chemoresistance to 5-FU in CRC cells by suppressing Hippo signaling *via* the transcriptional activation of ROCK1 and ROCK2 (26). Rho family mechano-signaling through the actin cytoskeleton positively regulates physiological TEAD/YAP transcription, with ROCK1 and ROCK2 acting as key effectors that enhance YAP activity. These findings suggest a potential connection between TFAP2C and YAP (27, 28). However, the specific mechanism by which TFAP2C transcriptionally activates Yes-associated protein (YAP) to promote cisplatin resistance in bladder cancer has not been previously reported, highlighting an innovative area for future research.

These findings build upon previous research that identified YAP and β-catenin as key mediators of cisplatin resistance in bladder cancer. Prior studies have shown that overexpression of YAP contributes to chemoresistance by enhancing DNA repair mechanisms and promoting cell survival, while inhibition of YAP can sensitize cancer cells to chemotherapy (7). Similarly, activation of the Wnt/β-catenin signaling pathway has been implicated in promoting tumor proliferation and resistance to apoptosis, contributing to chemoresistance (15). This study advances the field by identifying TFAP2C as an upstream regulator of YAP, directly binding to its promoter and enhancing its transcription, which in turn promotes β-catenin expression. While previous research focused on the downstream effects of YAP and β-catenin, this study elucidates a novel mechanism involving TFAP2C's role in regulating these pathways, representing a significant progression in understanding cisplatin resistance in bladder cancer.

Looking forward, future research could explore the therapeutic potential of targeting TFAP2C in combination with existing chemotherapeutic agents to overcome cisplatin resistance. Investigating whether TFAP2C inhibition can sensitize resistant bladder cancer cells to other treatments, such as immunotherapy or targeted therapies, could provide valuable insights. Additionally, since the current study primarily utilized cell lines and xenograft models, validating these findings in clinical samples and patient-derived organoids would enhance their translational relevance. Further studies might also examine the interaction between TFAP2C and other transcription factors or non-coding RNAs that may modulate its activity. Modifying experimental conditions, such as exploring the effects of tumor microenvironment factors like hypoxia or varying extracellular matrix components, could uncover additional layers of regulation and identify new targets for intervention, ultimately contributing to more effective treatment strategies for patients with bladder cancer.

## Experimental procedures

### Data source

Gene expression and clinical data for bladder cancer (BC) were gained from TCGA *via* the UCSC Xena platform (https://xenabrowser.net/). Including RNA sequencing data from 414 BC samples and 19 normal samples. The GSE231835 dataset (https://www.ncbi.nlm.nih.gov/geo/query/acc.cgi?acc=GSE231835), obtained from the Gene Expression Omnibus (GEO) database, was used to analyze the gene expression profiles of the T24 bladder cancer cell line and its cisplatin-resistant derivative, T24R2.

### Prognostic analysis

Using data from the TCGA-BLCA cohort, a prognostic analysis of YAP, FOXA1, and TFAP2C expression in bladder cancer (BC) was conducted. Patients were stratified into high- and low-expression groups based on median or quartile expression levels. Kaplan-Meier survival curves were generated to evaluate overall survival (OS) and disease-free survival (DFS), with statistical differences between groups assessed through the log-rank test. To determine the influence of YAP expression and other clinical variables on patient survival, Cox proportional hazards regression analysis was performed, considering *p* < 0.05 as statistically significant.

### Analysis of differential expression

Differential expression on GSE231835 datasets was analyzed by the R package limma. Differential gene expression was determined using the edgeR package in R, applying the criteria of adjusted *p* < 0.05 and | log_2_FC | >1.

### Upstream transcription factors (TFs) identification

The KnockTF v2 database (https://bio.liclab.net/KnockTFv2/index.php), which provides transcription factor-target interactions based on genome-wide CRISPR screening data, was used to identify TFs. A total of 125 TFs that may regulate YAP expression were retrieved.

### Correlation analysis

Pearson correlation analysis was performed using RNA-seq data from the TCGA-BLCA dataset to assess the relationship between YAP expression and 61 transcription factors. Expression data for YAP and transcription factors were extracted, and Pearson correlation coefficients (r) were calculated for each pair. Transcription factors with significant correlations (*p* < 0.05) were identified, with 13 showing a negative correlation and 48 showing a positive correlation with YAP expression.

### Clinical samples

Clinical samples from 32 BC patients were collected, including both tumor tissues and adjacent normal tissues. Clinical data are provided in Table S1. Following surgical resection, all specimens were stored at −80 °C for RNA and protein extraction. Written informed consent was obtained from all patients or their families. The study abided by the Declaration of Helsinki principles and was approved by the ethics committee of Jiangxi Medical College's First Affiliated Hospital, approval number: (2024) CDYFYYLK (07-037).

### Cell culture

Eight human bladder cancer cell lines (T24, 5637, J82, RT4, UM-UC-3, SW780, HT-1376, and TCCSUP) and Human bladder epithelial cell line SV-HUC-1 were sourced from the American Type Culture Collection (ATCC). They were cultured in RPMI-1640 medium (#11875-093, Gibco) supplemented with 10% fetal bovine serum (FBS, #26140-079, Thermo Fisher Scientific) and 1% penicillin-streptomycin solution (#SV30010, HyClone). The cells were maintained in a humidified incubator at 37 °C with 5% CO_2_ and passaged when they reached 80 to 90% confluence.

### Quantitative real-time PCR (qRT-PCR)

Total RNA was isolated from clinical samples and cell lines using the RNeasy Mini Kit (74,104, Thermo Fisher Scientific) following the manufacturer's protocol. RNA concentration and integrity were evaluated before subsequent steps. Reverse transcription was carried out with the High-Capacity cDNA Reverse Transcription Kit (4,368,814, Thermo Fisher Scientific) to synthesize cDNA. The resulting cDNA served as a template for quantitative PCR, performed using SYBR Green Master Mix (4,309,155, Thermo Fisher Scientific) as the fluorescent dye. The condition of the qPCR as follows: 95 °C: 30 s; 55 to 65 °C: 30 s; 72 °C: 1 min; Repeat for 30 cycles (total amplification cycles). Gene expression levels were quantified using the 2-ΔΔCt method, with β-actin used as the internal reference gene to normalize the expression of TFAP2C and YAP. Primer sequences are provided in Table 1.

**Table 1:** Primer sequences of the target genes

Genes	Primer	Primer sequences
TFAP2C	Forward	5′- GAGGAGGACTTCCTGCTCAG -3′
	Reverse	5′- CCATGTCCAGCTTCCTGTTC -3′
YAP	Forward	5′- CAGCCAGCCAGATGAGAAGA -3′
	Reverse	5′- GGGTGTATGGTGTGGTTGGT -3′
β-actin	Forward	5′-GAGACCTTCAACACCCCAGC-3′
	Reverse	5′-AGGAAGGAAGGCTGGAAGAG-3′

### Western blot

Clinical tissue samples and cell lysates were prepared using RIPA lysis buffer (P0013B, Beyotime, China). Total protein concentrations were measured, and equal amounts of protein were resolved by SDS-PAGE before being transferred onto PVDF membranes. The membranes were blocked with 5% non-fat milk and subsequently incubated with primary antibodies, including YAP (#A18428, ABclonal, 1:1000), p-YAP (#A5844, ABclonal, China, 1:1000), TFAP2C (#A5430, ABclonal, 1:1000), and β-catenin (#A5222, ABclonal, China, 1:1000). Following primary antibody incubation, the membranes were treated with an HRP-conjugated secondary antibody (#AS014, ABclonal, 1:5000). Protein bands were visualized using an ECL detection system.

### Cell transfection

Cell transfection was performed using Lipofectamine 3000 (#L3000015, Invitrogen, USA) according to the manufacturer's instructions. sh-TFAP2C plasmid (GeneChem, China) was used to knock down TFAP2C expression. The sequences of siRNAs and shRNA to TFAP2C are provided in Table 2. UM-UC-3 and T24 bladder cancer cells were seeded at 1 × 10^6^ cells per well in 6-well plates. Transfection was conducted when cells reached 60 to 70% confluence. Cells were collected for subsequent analysis after 48 h. qRT-PCR was used to verify the transfection efficiency.

**Table 2:** The sequences of siRNAs and shRNA to TFAP2C

Names	Sense strand	Scrambled control
siRNA 1	CCTCAGCUCUACGUCUAAAUA	GCUACGUAUCAGCGUACUAGA
siRNA 2	AUGGAGAAACACAGGAAAUAA	CGAUAGUACUGCGAUAGUACU
shRNA	ATGGAGAAACACAGGAAATAATTCAAGAGATTATTCCGTGTTTCTCCATG	GTACTCGAACGTAGTCTTCAATTCAAGAGATTGAAGACTACGTTCGAGTAC

### Establishment of cisplatin-resistant cell line

Gradually exposing T24 cells to increasing concentrations of cisplatin to establish the Cisplatin-resistant T24 bladder cancer cells (T24-R) (#P4394, Sigma-Aldrich, USA). Initially, T24 cells were treated with 0.1 μM cisplatin for 24 h, and the concentration was gradually increased by 0.1 μM every 3 days until the cells were able to survive at 10 μM cisplatin, which was the final concentration used to establish the resistant cell line. The cells were cultured in RPMI-1640 medium supplemented with 10% FBS and 1% penicillin-streptomycin solution at 37 °C in a 5% CO_2_ incubator. After the establishment of the resistant cell line, the cells were maintained in the same medium containing 10 μM cisplatin for continuous selection. The resistance phenotype was confirmed by performing cell viability assays using a CCK-8 kit (#CK04, Dojindo) to assess cell survival at increasing cisplatin concentrations.

### CCK-8 assay

Cells were plated in 96-well plates at a density of 5 × 10^3^ cells per well. Following treatment for 24, 48, 72, and 96 h, Added 10 μl of CCK-8 solution to each well and incubated at 37 °C for 2 h. Absorbance at 450 nm was measured using a microplate reader (Multiskan GO, Thermo Fisher).

To determine the IC50 of cisplatin, cells were seeded at the same density (5 × 10^3^ cells per well) in 96-well plates and cultured for 24 h. Subsequently, the cells were exposed to various concentrations of cisplatin for 48 h. After treatment, 10 μl of CCK-8 reagent was added to each well, and the plates were incubated at 37 °C for 2 h. The absorbance at 450 nm was recorded using a microplate reader, and the IC_50_ value was calculated based on the dose-response curve.

### Sphere formation assay

Tumor spheroid formation was performed using the hanging drop method. Briefly, 2 × 10^3^ tumor cells were resuspended in 100 μl of serum-free medium and seeded in hanging drops on the inner surface of a Petri dish lid. The lid was inverted, and the dish was placed in a humidified incubator at 37 °C with 5% CO_2_ for 7 days to allow spheroid formation. The medium was replenished every 2 to 3 days. Spheroid morphology and size were observed under an inverted microscope. For visualization, spheroids were stained with Calcein-AM (#C3099, Invitrogen, USA) for live-cell imaging.

### Cell apoptosis

BC cells were collected and stained with Annexin V-FITC/PI according to the instructions of the Annexin V-FITC/PI Apoptosis Detection Kit (#K101-25; BioVision). The fluorescence intensity was detected by BD FACSCanto II Flow Cytometer (BD Biosciences), and the data analysis was performed by FlowJo software (v10.6).

### Transwell assay

1 × 10^4^ BC cells were seeded into the upper chamber of a Transwell insert (#3422, Corning) coated with Matrigel. The lower chamber was filled with 600 μl of complete medium as a chemoattractant. After incubation for 24 h at 37 °C with 5% CO_2_, cells that migrated or invaded to the lower surface of the membrane were fixed with 4% paraformaldehyde (#P6148, Sigma-Aldrich) and stained with crystal violet (#C6158, Sigma-Aldrich). Several fields were randomly selected under the microscope (Nikon Eclipse Ti2) for imaging and counting.

### ChIP-qPCR

UM-UC-3-R and T24-R cells were cross-linked with 1% formaldehyde and lysed. Chromatin was sheared by sonication to an average size of 200 to 1000 bp. For each ChIP reaction, 5 μg of chromatin DNA was used for both the TFAP2C immunoprecipitation and input control. Immunoprecipitation was performed using anti-TFAP2C antibody (#H00007022-M01, Invitrogen) or an isotype control IgG antibody (#A7016, Beyotime, China). After reversal of cross-links and DNA purification, qPCR was performed using primers specific to the YAP promoter region and an additional primer pair targeting a distal region ∼1.8 kb upstream of the YAP transcription start site. The sequences of the distal control primers were: Forward: 5′-AGACAGGAAACTGGGGGTTC-3′; Reverse: 5′-CACTTGGTGTGGTAGGGCTC-3′. Enrichment was analyzed by comparing Ct values of immunoprecipitated samples with input controls.

### Dual-luciferase reporter assay

UM-UC-3-R and T24-R bladder cancer cells were co-transfected with a pGL3-basic vector containing the YAP promoter region and either an empty vector or a TFAP2C expression plasmid, along with a Renilla luciferase control plasmid (#E2261, Promega) for normalization. After 48 h, cells were harvested and lysed, and luciferase activity was measured using the Dual-Luciferase Reporter Assay System (#E1910, Promega) following the manufacturer's instructions. The ratio of Firefly (YAP promoter activity) to Renilla luciferase activity was calculated to determine the effect of TFAP2C on YAP transcriptional activation.

### YAP promoter deletion luciferase assay

A series of deletion luciferase reporter constructs (P-Δ1–P-Δ5) covering the −2000 to 0 bp region upstream of the YAP coding sequence were generated and cloned into the pGL3-basic vector. These constructs were co-transfected with a TFAP2C overexpression plasmid and a Renilla luciferase internal control plasmid (pRL-TK, Cat# D2760, Beyotime) into 293T cells. After 48 h, luciferase activity was measured using the Dual-Luciferase Reporter Assay Kit (Cat# RG027, Beyotime) according to the manufacturer's instructions. The ratio of Firefly to Renilla luciferase activity was calculated to assess the contribution of each promoter region to TFAP2C-mediated transcriptional activation.

### Subcutaneous tumor mouse model

6-week-old female BALB/c-nu/nu nude mice were obtained from Shanghai SLAC Laboratory Animal Co., Ltd and housed in SPF-grade facilities at 24 ± 1 °C, 50 to 60% humidity, with a 12-h light/dark cycle. All procedures complied with NIH Guidelines for the Care and Use of Laboratory Animals and were approved by the Jiangxi Medical College's First Affiliated Hospital (Approval No.: CDYFY-IACUC-202407QR024). Mice (n = 10 per group) were divided into three groups: Cisplatin-sensitive group, Cisplatin-resistant group, and TFAP2C-silenced resistant group. Mice were subcutaneously injected with 1 × 10^6^ cells in the right axilla: T24 cells for the Cisplatin-sensitive group, T24-R cells for the Cisplatin-resistant group, and TFAP2C-silenced T24-R cells for the TFAP2C-silenced resistant group. Cisplatin was administered *via* intraperitoneal injection at a dose of 5 mg/kg once weekly for a total of 6 weeks. Tumor growth was monitored weekly by measuring the tumor's longest and shortest diameters with a caliper, and tumor volume was calculated using the formula: volume= (width ^2^ × length)/2. At the experimental endpoint, tumors were excised, and their weights were measured.

### Statistical analysis

Statistical analysis was performed by GraphPad Prism version 9.5.1. All experiments were repeated at least 3 times and results were presented as mean ± SD. Significance (*p* < 0.05) between two groups was evaluated according to Student's *t* test. Two-way ANOVA was performed to evaluate the effects of both factors and their interaction on tumor volume or other outcomes.

## Data availability

The GEO datasets was publicly available (https://www.ncbi.nlm.nih.gov/geo/query/acc.cgi?acc=GSE231835). The data generated and analyzed during the current study are available from the corresponding author upon reasonable request.

## Supporting information

This article contains supporting information.

## Ethical approval

This study was conducted in accordance with the Declaration of Helsinki and approved by the Ethics Committee of [Jiangxi Medical College's First Affiliated Hospital], approval number: [(2024) CDYFYYLK (07-037)]. Written informed consent was obtained from all participants or their legal guardians.

## Conflict of interest

The authors declare that they have no conflicts of interest with the contents of this article.
