# Supplementary material for: TFAP2C drives cisplatin resistance in bladder cancer by upregulating YAP and activating β-catenin signaling
Source: J Biol Chem. 2025 Jun 18;301(8):110387. doi: 10.1016/j.jbc.2025.110387 (PMC12284524; doi:10.1016/j.jbc.2025.110387)
Supplement: Supplementary Tables [file mmc1.docx]

**Supplementary Tables**

**Table S1.** Clinical data of 32 BC patients.

| **Case** | **Gender** | **Age** | **T** | **N** | **M** |
| --- | --- | --- | --- | --- | --- |
| 1 | MALE | 49 | T4 | N2 | M0 |
| 2 | FEMALE | 61 | T1 | N0 | M0 |
| 3 | MALE | 70 | T3 | N0 | M0 |
| 4 | MALE | 67 | T2 | N0 | M0 |
| 5 | MALE | 70 | T1 | N0 | M0 |
| 6 | MALE | 67 | T1 | N0 | M0 |
| 7 | MALE | 73 | T4 | N1 | M0 |
| 8 | MALE | 73 | T2 | N0 | M0 |
| 9 | MALE | 77 | T2 | N2 | M0 |
| 10 | MALE | 70 | T4 | N1 | M0 |
| 11 | MALE | 79 | T3 | N0 | M0 |
| 12 | MALE | 39 | T4 | N1 | M0 |
| 13 | FEMALE | 86 | T4 | N1 | M0 |
| 14 | FEMALE | 84 | T3 | N2 | M0 |
| 15 | MALE | 71 | T3 | N1 | M0 |
| 16 | MALE | 67 | T2 | N0 | M0 |
| 17 | MALE | 58 | T2 | N2 | M0 |
| 18 | MALE | 87 | T3 | N0 | M0 |
| 19 | MALE | 59 | T2 | N0 | M0 |
| 20 | MALE | 57 | T1 | N0 | M0 |
| 21 | MALE | 77 | T4 | N0 | M0 |
| 22 | MALE | 77 | T2 | N0 | M0 |
| 23 | MALE | 61 | T4 | N2 | M0 |
| 24 | MALE | 49 | T2 | N0 | M0 |
| 25 | MALE | 73 | T4 | N2 | M0 |
| 26 | MALE | 53 | T4 | N2 | M0 |
| 27 | MALE | 67 | T4 | N2 | M0 |
| 28 | FEMALE | 65 | T3 | N0 | M0 |
| 29 | FEMALE | 72 | T3 | N0 | M0 |
| 30 | MALE | 69 | T4 | N2 | M0 |
| 31 | MALE | 69 | T4 | N0 | M0 |
| 32 | MALE | 74 | T2 | N0 | M0 |

**Table S2.** 61 TFs showed significant correlations with YAP, with 48 positively correlated and 13 negatively correlated.

| **Correction with YAP Gene** | |
| --- | --- |
| **Positive** | **Negative** |
| CNOT7 | SNRNP70 |
| USP22 | SAFB2 |
| LHX9 | PRMT1 |
| SIX4 | FOXA1 |
| FOXK2 | ESRRG |
| ERF | UBE2L3 |
| NKRF | CCAR2 |
| TWIST1 | HOXC6 |
| FOXM1 | SMARCA4 |
| RBM15 | TFAP4 |
| FOXO1 | SOX4 |
| AGO1 | HMGN3 |
| TWIST2 | MYB |
| POSTN |  |
| HNRNPLL |  |
| SOX9 |  |
| ARID1A |  |
| PLAG1 |  |
| SMG7 |  |
| SMARCA2 |  |
| BCLAF1 |  |
| SALL4 |  |
| EHF |  |
| SOX7 |  |
| E2F7 |  |
| MYC |  |
| SRF |  |
| PRPF4 |  |
| NMI |  |
| STRAP |  |
| SP1 |  |
| ERG |  |
| MEF2D |  |
| RUNX1 |  |
| NFE2L1 |  |
| SNW1 |  |
| TCF7L1 |  |
| CREBBP |  |
| MITF |  |
| SMARCA1 |  |
| PCBP1 |  |
| ZFP36L1 |  |
| TFAP2C |  |
| ACTN4 |  |
| STAT3 |  |
| SP3 |  |
| HIF1A |  |
| ELK3 |  |

**Table S3.** Differential expression analysis of TFs in the GSE231835 dataset in the cisplatin-resistant samples compared to controls (3 TFs were significantly upregulated, while 18 were significantly downregulated ).

|  | Gene | baseMean | Log FC | P.Value | adj.P.Val |
| --- | --- | --- | --- | --- | --- |
| Upregulated | TFAP2C | 230.999394072315 | 2.45366624003981 | 7.00015833550269e-107 | 2.72519592198725e-105 |
|  | GLI1 | 13.9900468674334 | 1.3976232436064 | 6.11981367892998e-05 | 0.000164210113773815 |
|  | AR | 15.6531477788299 | 1.0577931222868 | 0.000729004543026368 | 0.00172126654726442 |
| Downregulated | IKZF2 | 93.311514928727 | -1.16103002 | 1.09291947760135e-13 | 5.40493973239615e-13 |
|  | SALL4 | 53.6766429752368 | -4.069111603 | 2.39722143816519e-40 | 3.04352024217953e-39 |
|  | SMARCA1 | 796.427786585511 | -4.808957615 | 0 | 0 |
|  | TLE4 | 249.091192614633 | -2.030336709 | 3.93602522495692e-84 | 1.14787538010193e-82 |
|  | GATA3 | 111.014152243519 | -6.549009367 | 2.8189326598152e-53 | 4.75150188343543e-52 |
|  | BCL6 | 163.192876914674 | -1.171719846 | 3.16282929036422e-19 | 2.02920078631725e-18 |
|  | FOXP1 | 285.7702643 | -1.185519279 | 6.57859482469053e-44 | 9.0770611570464e-43 |
|  | MYB | 8.27419300977474 | -1.186205373 | 0.00137921779226265 | 0.0031319631454781 |
|  | SOX4 | 516.964874380404 | -2.432255509 | 5.74058508003223e-173 | 4.33942448243049e-171 |
|  | FOXA1 | 400.490266386284 | -2.269064414 | 5.12174913898629e-165 | 3.63732749371871e-163 |
|  | POSTN | 5.53972218862293 | -3.602990097 | 2.82745088964117e-05 | 7.83590556381324e-05 |
|  | EHF | 34.3929670852657 | -5.748412173 | 5.7429017791847e-21 | 3.95977416184803e-20 |
|  | HMGA1 | 27150.9939100666 | -1.243667475 | 6.89582540331777e-207 | 6.79735301655839e-205 |
|  | HNRNPLL | 121.80089544224 | -1.564561054 | 2.6358186411474e-32 | 2.6686310096054e-31 |
|  | FOXO1 | 275.151597825503 | -2.041056356 | 2.1638625522284e-102 | 7.88817527730243e-101 |
|  | FOXQ1 | 12.2710664552798 | -2.149846953 | 1.54463791089217e-07 | 5.23367414246057e-07 |
|  | E2F7 | 933.844380456052 | -1.376207709 | 2.58366592888547e-77 | 6.73748989264812e-76 |
|  | SOX7 | 34.7912591663726 | -1.469684585 | 5.76798258134016e-09 | 2.17140841356501e-08 |
